# Evaluation and Comparison of Mucormycosis Patients' Features Undergoing Functional Endoscopic Sinus Surgery Prior to and during the COVID-19 Pandemic: A Case-Control Study

**DOI:** 10.1155/2022/1248325

**Published:** 2022-06-07

**Authors:** Laleh Dehghanpisheh, Mohammadhossein Eghbal, Mehrdad Salari, Reza Shahriarirad, Niloofar Borzou, Pooya Vatankhah

**Affiliations:** ^1^Anesthesiology and Critical Care Research Center, Department of Anesthesiology and Critical Care, School of Medicine, Shiraz University of Medical Sciences, Shiraz, Iran; ^2^Thoracic and Vascular Surgery Research Center, Shiraz University of Medical Science, Shiraz, Iran

## Abstract

**Background:**

Rhinocerebral mucormycosis is a serious invasive fungal infection that is one of the most aggressive and lethal of invasive mycoses. The coronavirus disease (COVID-19) has been linked to immune dysregulation, and patients with COVID-19 have been reported to be at risk for developing invasive fungal infections. This study is conducted to evaluate the concurrence of mucormycosis among COVID-19 patients.

**Methods:**

In this retrospective, cross-sectional study, hospital records of patients with mucormycosis, as well as COVID-19 admitted to Khalili Hospital, as the major referral center for functional endoscopic sinus surgery (FESS) in southern Iran, were collected. Demographic and clinical information was extracted and subsequently analyzed.

**Results:**

Among 59 mucormycosis patients undergoing FESS, 41 (69.5%) were during the COVID-19 pandemic, while 18 (30.5%) were during one year before the COVID-19 pandemic. The average age was 49.33 ± 20.52, and 64.4% had diabetes mellitus, while 62.7% had COVID-19. The most common presentation was periorbital edema (56.9%), followed by necrotic tissue (48.3%). Although the total number of cases increased during the COVID-19 period compared to the case before the pandemic, the overall pattern and features of the patients had no significant difference, except regarding a significant increase in the presentation of necrotic tissue and also the use of corticosteroids. Most cases developed mucormycosis two weeks after COVID-19. The overall mortality was 36.8%, which is not statistically associated with COVID-19.

**Conclusion:**

Even in the absence of comorbidities, physicians should be aware of the risk of secondary fungal infections in patients with COVID-19 who were treated with corticosteroids.

## 1. Background

Mucormycosis is an invasive fungal infection that most commonly affects diabetic and immunocompromised patients, particularly in its rhino-orbital-cerebral or pulmonary forms. Rarely, mucormycosis may involve the trachea and main bronchi [1,2]. Mucormycosis is an opportunistic infection that usually involves diabetic patients (with or without ketoacidosis), cases with hematologic malignancies or organ transplantation, and patients who are on iron chelator or broad-spectrum antibiotics. The most common form of pulmonary involvement in mucormycosis is pneumonic infiltration with variable and somewhat characteristic radiologic features [1,2]. The involvement of large airways with or without parenchymal disease is less common [1,2].

Rhinocerebral mucormycosis is a serious invasive fungal infection that is one of the most aggressive and lethal of invasive mycoses. Uncontrolled diabetes, organ transplant, malignancies such as lymphoma and leukemia, immunosuppressive therapy, renal failure, and acquired immune deficiency syndrome (AIDS) are all underlying diseases for mucormycosis [3]. Disseminated rhino-orbital-cerebral mucormycosis is a lethal invasive fungal infection that accounts for 8.3–13 percent of all fungal infections found in hematological patients' autopsies. Hematological malignancy, immunosuppressed children, and Diabetic ketoacidosis (DKA) are all predisposing factors. Uncontrolled diabetes mellitus, periorbital infection, and meningoencephalitis are all part of the mucormycosis triad [4].

The ongoing COVID-19 outbreak began in December 2019 in Wuhan, China. COVID-19, the disease linked to SARS-CoV-2 infection, spread quickly, resulting in a global pandemic [5]. The severity of symptoms associated with SARS-CoV-2 infection ranges from mild to severe [6, 7]. The proportion of infections that are severe or fatal varies by location. COVID-19 patients may have elevated levels of inflammatory cytokines (such as interleukin [IL]-2R, IL-6, IL-10, and tumor necrosis factor-alpha), as well as a weakened cell-mediated immune response, affecting both CD4+ T and CD8*+ T* cells. As a result, there is an increased susceptibility to fungal coinfections [8]. Also, comorbid diseases and conditions such as immunodeficiency, diabetes, and transplantation can act as a risk factor for both COVID-19 and other types of bacterial or fungal infections [7, 9–12]. COVID-19 and acute respiratory failure are treated with a combination of broad-spectrum antibiotics and corticosteroids (both of which are risk factors for invasive fungal disease) [13]. COVID-19 has been linked to immune dysregulation [14], and patients with COVID-19 have been reported to be at risk for developing invasive fungal infections like invasive aspergillosis, candidiasis, and *Pneumocystis jiroveci* infection [15, 16]. However, the consequents of the impact of the COVID-19 pandemic, along with diagnostic and management challenges, remain [17–22].

Infarction and necrosis of the host tissues are symptoms of mucormycosis, which is caused by hyphae invading the vasculature. Mucormycosis can present as a variety of syndromes depending on the anatomic site involved, including rhino-orbital-cerebral, pulmonary, cutaneous, and less commonly GI, renal, and disseminated diseases [23].

Surgical debridement of the affected tissues and antifungal therapy is used to treat the condition. The drug of choice for initial therapy is intravenous amphotericin B (a lipid formulation) [24]. The prognosis for recovery from mucormycosis is poor despite early diagnosis and aggressive combined surgical and medical therapy [25]. Effective management consists of cytological diagnosis, optimization of predisposing conditions, systemic antifungal therapy with prompt, and surgical debridement of infected tissue, via methods such as functional endoscopic sinus surgery (FESS) [4].

The ability to cure the condition depends on clinical suspicion and early therapy. As a result, if symptoms imply mucormycosis, preemptive medication should be explored. More studies are necessary to determine if these two pathologies are related. Therefore, this study is conducted to evaluate the features of patients who underwent FESS due to mucormycosis and its concurrence with COVID-19 patients in Southern Iran.

## 2. Material and Methods

In this retrospective, cross-sectional study, all patients who underwent FESS due to mucormycosis and had not responded to medical treatment from April 2020 till September 2021 were included in our study. Patients with the diagnosis of mucormycosis were initially administered intravenous liposomal amphotericin B 5–10 mg/kg/day, followed by step-down therapy with Posaconazole [24, 26]. Patients who did not have sufficient response to medical treatment, such as the persistence or progression of sign and symptoms, or positive necrosis in their biopsy were scheduled for FESS. Data of these patients were retrieved from the archives of Khalili Hospital, which is the major referral center in southern Iran for FESS. Hospital records extraction was carefully based on the specific disease code. Demographic information, case number, and place of residence and information related to disease, age, sex, length of hospital stay, symptoms, underlying disease, hospital stay, need for intubation, and need for chest tube insertion of patients with mucormycosis, were extracted, and information regarding COVID-19 (based on clinical symptom criteria, chest CT scan, and positive report of COVID-19 test based on Rt-PCR SARS-CoV-2) was used for the extraction, where this information was subsequently statistically analyzed.

The present study was approved by the Medical Ethics Committee of Shiraz University of Medical Sciences. The study was conducted in compliance with local regulatory requirements, Good Clinical Practice (GCP), and the Declaration of Helsinki and according to the STROBE checklist. Due to the retrospective nature of our study, inform consent was not obtained from the patients, and the Ethics committee of Shiraz University of Medical Sciences waived the need for informed consent. The patients' information was documented from their hospital records. Patients' data were anonymized prior to analysis, and their confidentiality was secured by the researcher. All study protocols and data collection were performed in accordance with the Declaration of Helsinki and approved by the mentioned ethics committee.

The data was analyzed using IBM Corporation's SPSS software version 22 (Armonk, NY). The Kolmogorov-Smirnov normality test was used to determine whether the data were normal. Data analysis was carried out using independent sample students' *t*-test and ANOVA test for continuous variables and chi-square for categorical variables after validation of the parameters' normal distribution. Frequency, percentage, mean data distribution, and standard deviation are components of descriptive statistics (SD).

## 3. Results

**Figure 1:**
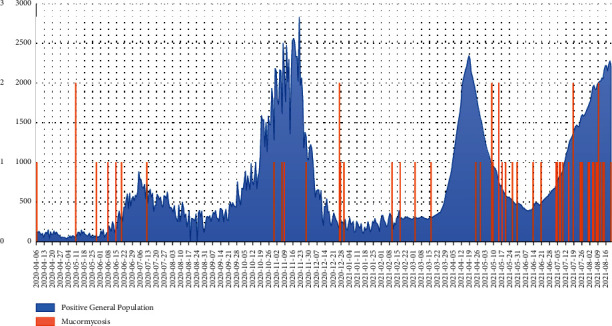
Frequency of mucormycosis cases in Khalili hospital (a referral hospital in Shiraz, Fars, Iran) based on the total positive COVID-19 cases among the general population in Fars province, southern Iran.

During the period of our study, a total of 59 cases of mucormycosis who underwent FESS were documented. Among the patients, 41 (69.5%) were during the COVID-19 pandemic, while 18 (30.5%) were during one year before the COVID-19 pandemic.  Figure 1 demonstrates the frequency of cases during the timeline of our study, along with a comparison with the total number of COVID-19 cases in our study area.

Among the patients, 55 (93.2%) had comorbid diseases, in which diabetes mellitus was the most prevalent comorbidity among our patients. The patients' age ranged from 2.5 years to 80 years old. The overall features of the patients are demonstrated in Table 1. Also, 37 (62.7%) of our patients had COVID-19.

The most common presentation in our patients was periorbital edema (56.9%), followed by necrotic tissue (48.3%). Also, the most common comorbidity was diabetes mellitus (64.4%). As demonstrated in Table 1, based on the two timelines in our study, although the total number of cases increased during the COVID-19 period compared to the case before the pandemic, the overall pattern and features of the patients had no significant difference, except regarding a significant increase in the presentation of necrotic tissue, and also the use of corticosteroids (*P*=0.003 and 0.001, respectively). While comparing the COVID-19 group with the noninfected group, there was only a significantly higher number of diabetic patients with COVID-19, compared to non-COVID-19 diabetic patients (*P*=0.026). Also, COVID-19 patients demonstrated a significantly higher frequency of necrotic tissue compared to the non-COVID-19 group (*P*=0.016).

The patient's hospitalization features are demonstrated in Table 2. Based on CT-scan evaluation, 24 (64.9%) of the patients had features in favor of COVID-19, while 2 (5.4%) of the COVID-19 patients had normal CT scans.

We evaluated the hospitalization and illness course of the patients in our study (Table 2). The average hospitalization duration following FESS did not change during the COVID-19 pandemic, while also there was no statistically significant difference regarding the patients' vital signs based on COVID-19.

Regarding the radiological evaluation of the patients, the most frequent finding in the PNS CT was mucosal thickening (51.7%) followed by sinusitis (48.3%), which did not alter during and prior to the COVID-19 pandemic or among COVID-19 positive and negative patients.

Based on laboratory evaluation, there were no significant differences regarding white blood cell count and differentiation or hemoglobulin count based on COVID-19 among our patients. However, COVID-19 patients had significantly lower platelet counts compared to the non-COVID-19 group (*P* < 0.001).

Regarding ventilation, the majority of our patients did not require any supportive ventilation (64.9%), which was also similar among the COVID-19 and non-COVID-19 groups; however, 10.5% of the patients required intubation, which also was not related to COVID-19.

Based on the performed operation among our patients, 19 (32.2%) required additional operations such as maxillectomy, orbital exenteration, and frontal lobectomy, aside from FESS. Also, the mortality rate in our study was 21 (36.8%) cases, which was unrelated to COVID-19. There was also no significant association between the duration of symptoms and the patient's outcome (*P*=0.685).

As demonstrated in Table 2, the number of patients presenting with mucormycosis on admission was significantly higher among COVID-19 patients (*P*=0.040). Most cases developed mucormycosis two weeks after COVID-19.

## 4. Discussion

Early identification, management of the underlying disease, administration of antifungal medication (such as amphotericin B), radical surgical debridement, and other adjuvant treatments are all important factors in improving rhinocerebral mucormycosis survival [27–29]. Early diagnosis is critical because it allows for more rapid implementation of appropriate treatment [30]. Early diagnosis relies on the patient seeking medical treatment as soon as possible, the physician's suspicion of the condition, and the pathologist's definitive confirmation of the diagnosis [31]. When a diagnosis is delayed, it is impossible to provide prompt and effective therapy. The most challenging and crucial component of treatment may be maintaining control of the underlying condition. When a number of predisposing variables play a role in the development of rhinocerebral mucormycosis, early detection and treatment of these factors are critical for survival. The prognosis differs depending on the underlying condition [27]. Diabetes is linked to a higher percentage of survival than nondiabetic underlying diseases. Diabetes mellitus was the underlying illness in the majority of our patients, similar to other reports [32]. Recent studies have also focused on COVID-19, as a predisposing factor for mucormycosis [33,34]. The pathogenesis of mucormycosis shows that normal hosts' mononuclear and polymorphonuclear phagocytes kill Mucorales by producing oxidative metabolites and defensins, so neutropenic patients and those with dysfunctional phagocytes are at risk of developing invasive mucormycosis [35,36]. There is profound lymphopenia in COVID-19, and viral replication exacerbates the inflammatory response and neutrophil and monocyte influx in the bloodstream in advanced infections [37]. As a result of the imbalance in neutrophil and lymphocyte activity, the patient becomes more susceptible to systemic fungal infections.

Similar to our results, a study in India reported a significant increase (nearly fourfold) in the number of FESS cases in their institution for mucormycosis removal, when comparing the last two years to the year 2021 [38]. FESS has been reported to be effective in the treatment of fungal ball. Surgery prevents endocranial complications in cases of fulminant invasive mycoses.

During the first wave in Iran, we believe that there was significant underreporting, and there was a surge of cases in the medical community during the first wave as well. The rise in mucormycosis cases, on the other hand, was interpreted as a coincidence rather than an outbreak. Due to the massive increase in cases, widespread media attention, and coverage that occurred during the following waves, we see an increase in the number of mucormycosis cases (Figure 1), which could be due to higher awareness and prompt investigation, which is also supported by a study in India [39]. Knowledge and awareness of the disease among both the general public and physicians can count as important factors for timely diagnosis and proper management of the disease [40–42]. Poorly controlled diabetes, excessive use of corticosteroids and possibly antibiotics, and environmental exposure may all play a role in the significantly higher prevalence of COVID-19-induced mucormycosis in Iran and also other countries [43]. Iran's hot and humid climate may have aided the growth of Mucorales species, as was the case in India [44].

As demonstrated in our results, most cases developed mucormycosis two weeks after COVID-19. The disease is most commonly seen during the COVID-19 recovery period, implying that several factors contribute to fungal colonization. The time between COVID-19 and the initial diagnosis of mucormycosis was 10 to 15 days in most of the cases in a study by Rao et al. in India [45]. Patients may have overlooked mucormycosis symptoms (especially pain), confusing them with residual COVID-19 symptoms, and thus arrived at the hospital late. Furthermore, dental symptoms should also be addressed during initial hospital visits, and nasal or sinus symptoms must be given priority with only magnetic resonance imaging (MRI). At a later stage of follow-up, patients may develop advanced maxillary disease (1–3 weeks) [45]. In order to limit tissue necrosis, it is critical to evaluate maxillary bone involvement on CT at an early stage.

A systematic review by Bhattacharyya et al. reported that COVID-19 causes a significant increase in mucormycosis in specific parts of the world. The overall mortality in our study was 36.8%, which is similar to other studies regarding orbital mucormycosis [46]. However, although most of our deceased cases had COVID-19, there was not any statistically significant association in this regard. COVID-19-associated mucormycosis death has been reported to be 16.3% based on a recent review by Muthu et al. [39].

Increased steroid use in COVID-19 patients could explain some of the increases in mucormycosis cases. Following the publication of the RECOVERY study's randomized-controlled trial [1], steroid use increased. Patients hospitalized with COVID-19 who were given dexamethasone had a lower 28-day mortality rate than those who were given invasive mechanical ventilation or oxygen alone, according to the study. Even though steroids have no benefit in patients who do not require respiratory support in the trial, many COVID-19 patients who do not require mechanical ventilation have been treated with glucocorticoids, even at higher doses and for longer periods than the trial recommended [44]. In a review by Dilek et al. [47], steroids were given to 90.5 percent of patients with mucormycosis and COVID-19. Another risk factor for mucormycosis is diabetes. Steroids, which aggravate hyperglycemia in patients with diabetes mellitus, are the most common cause of drug-induced hyperglycemia [48]. Hyperglycemia caused by diabetes is thought to impair immune response, making it difficult to control the spread of invading pathogens [49].

Tropical and subtropical humid climates, as well as high environmental temperatures in most parts of India, appeared to play a role in disease prevalence [44]. The COVID-19 pandemic added to the growing mucormycosis pandemic by introducing new risk factors. India is currently dealing with a new wave of the COVID-19, which has posed a threat to the country's healthcare system. India experienced another pandemic of mucormycosis during the COVID-19 pandemic.

Hypoxia of the tissues in COVID-19 disease is another factor that can play a role. The tissue damage is exacerbated by low oxygen levels in the tissues, as well as the partial infraction of fungal angioinvasion. Furthermore, the overuse of antibiotics, which is common in COVID-19 management, suppresses the normal bacterial flora, making it easier for fungi to establish and invade. Broad-spectrum antibiotic use is common in cases of COVID-19 with mucormycosis, according to a systematic review by Dilek et al. [47]. Antibiotics were used 74.6 percent of the time in COVID-19 cases, according to Langford et al. [50]. SEMI-COVID-19 analysis revealed that 78.1 percent of COVID-19 patients were prescribed antibiotics, with 34% of antibiotic prescriptions being inappropriate [51]. Despite the fact that antibiotic use has been shown to be ineffective, during the first wave of COVID-19 in India, an estimated 216 million excess antibiotic doses and 6.2 million azithromycin treatment courses were attributed to COVID-19 [44].

Recent studies suggest that COVID-19 is a procoagulable state with an increased risk of thrombotic events [37]. This procoagulable state is ideal for angioinvasion of *Mucor* invasion, which can lead to disseminated infections due to vessel thrombosis. Song et al. published a study in which they looked into a total of 99 patients who had fungal investigations after COVID-19 in China and discovered that about 5% of them were caused by *Aspergillus* species and 7% by *Mucor* species. They concluded that one of the most important pathogeneses is the impairment of T cell immunity in the presence of an underlying immunocompromised state [8]. Mehta and Pandey described a case of post-COVID-19 rhino-orbital mucormycosis, in which the patient received steroids according to protocol and developed mucormycosis as a result [52]. Regarding mucormycosis cases in our study, patients with COVID-19 had significantly lower platelet count compared to non-COVID-19 infected patients. According to their theory, alterations in immunity, particularly T cells and innate immunity, as well as the use of steroids, may be the cause of post-COVID-19 invasive fungal infection [52]. Amanda et al. from the United States and Chaudhary et al. from Delhi have made similar observations [53,54].

## 5. Conclusion

Even in the absence of comorbidities, physicians should be aware of the risk of secondary fungal infections in patients with COVID-19 who were treated with corticosteroids. Furthermore, the significance of a multidisciplinary approach should be taken into account.

## Figures and Tables

**Table 1:** Demographic and clinical features of patients.

Variable		Total;*N* = 59	Timeline		*P* value^*∗*^	COVID-19		*P* value^*∗*^
			Non-COVID-19 period; *n* = 18	COVID-19period;*n* = 41		Notinfected;*n* = 22	Infected;*n* = 37	
Age		49.33 ± 20.52	39.86 ± 25.69	53.49 ± 16.50	0.050	37.11 ± 24.57	56.59 ± 13.45	**0.002**

Gender	Male	37 (62.7)	10 (55.6)	27 (65.9)	0.451	11 (50.0)	26 (70.3)	0.166
	Female	22 (37.3)	8 (44.4)	14 (34.1)		11 (50.0)	11 (29.7)	

Comorbid disease	Diabetes mellitus	38 (64.4)	9 (50.0)	29 (70.7)	0.149	10 (45.5)	28 (75.7)	**0.026**
	Hypertension	31 (52.5)	7 (38.9)	24 (58.5)	0.257	8 (36.4)	2 (62.2)	0.065
	Cardiovascular	11 (18.6)	3 (16.7)	8 (19.5)	1.000	2 (9.1)	9 (24.3)	0.184
	Cerebrovascular Dx	10 (16.9)	3 (16.7)	7 (17.1)	1.000	2 (9.1)	8 (21.6)	0.294
	Malignancy	9 (15.3)	4 (22.2)	5 (12.2)	0.434	4 (18.2)	5 (13.5)	0.715
	Chronic kidney Dx	8 (13.6)	2 (11.1)	6 (14.6)	1.000	1 (4.5)	7 (18.9)	0.237
	Hypothyroidism	5 (8.5)	2 (11.1)	3 (7.3)	0.636	3 (13.6)	2 (5.4)	0.351
	Transplantation	5 (8.5)	1 (5.6)	4 (9.8)	1.000	2 (9.1)	3 (8.1)	1.000
	PTE or DVT	3 (5.1)	0 (0.0)	3 (7.3)	0.546	0 (0)	3 (8.1)	0.286
	Asthma	3 (5.1)	2 (11.1)	1 (2.4)	0.218	2 (9.1)	1 (2.7)	0.549
	Chronic liver Dx	2 (3.4)	0 (0.0)	2 (4.9)	1.000	1 (4.5)	1 (2.7)	1.000
	Chronic obstructive pulmonary Dx	1 (1.7)	0 (0.0)	1 (2.4)	1.000	1 (4.5)	0 (0)	0.373
	HIV	0 (0)	0 (0)	0 (0)	—	0 (0)	0 (0)	—
	None	4 (6.8)	3 (16.7)	1 (2.4)	0.080	3 (13.6)	1 (2.7)	0.141

Social history	Smoker	8 (13.6)	1 (5.6)	7 (17.1)	0.414	3 (13.6)	5 (13.5)	1.000
	Opium	2 (3.4)	1 (5.6)	2 (2.4)	0.521	0 (0.0)	2 (5.4)	0.524
	Waterpipe	1 (1.7)	0 (0)	1 (2.4)	1.000	1 (4.5)	0 (0.0)	0.373

Corticosteroid use	Yes	31 (53.4)	6 (33.3)	25 (62.5)	0.039	6 (27.3)	25 (69.4)	0.002
	COVID-19 related	19 (63.3)	0 (0)	19 (79.2)	**0.001**	0 (0)	19 (79.2)	**0.001**
	Non-COVID-19 related	11 (36.7)	6 (100)	5 (20.8)		6 (100)	5 (20.8)	

Physical exam, sign, and symptom	Periorbital edema	33 (56.9)	9 (52.9)	24 (58.5)	0.695	9 (40.9)	24 (66.7)	0.063
	Necrotic tissue	28 (48.3)	3 (17.6)	25 (61.0)	**0.003**	6 (27.3)	22 (61.1)	**0.016**
	Impaired vision or blindness	26 (44.8)	8 (47.1)	18 (43.9)	1.000	9 (40.9)	17 (47.2)	0.787
	Headache	23 (39.7)	3 (17.6)	20 (48.8)	0.039	6 (27.3)	17 (47.2)	0.171
	Frozen eye or loss of motion	10 (17.2)	2 (11.8)	8 (19.5)	0.707	2 (9.1)	8 (22.2)	0.290
	Facial edema	9 (15.5)	4 (23.5)	5 (12.2)	0.426	3 (13.6)	6 (16.7)	1.000
	Facial pain	9 (15.5)	1 (5.9)	8 (19.5)	0.258	2 (9.1)	7 (19.4)	0.459
	Eye pain	8 (13.8)	1 (5.9)	7 (17.1)	0.415	3 (13.6)	5 (13.9)	1.000
	Ptosis	8 (13.8)	0 (0.0)	8 (19.5)	0.090	1 (4.5)	7 (19.4)	0.139
	Eye proptosis	6 (10.3)	1 (5.9)	5 (12.2)	0.660	1 (4.5)	5 (13.9)	0.392
	Nasal discharge	5 (8.6)	3 (17.6)	2 (4.9)	0.144	3 (13.6)	2 (5.6)	0.357
	Blurred vision	4 (6.9)	2 (11.8)	2 (4.9)	0.573	2 (9.1)	2 (5.6)	0.630
	Ophthalmic ecchymosis	3 (5.2)	2 (11.8)	1 (2.4)	0.203	2 (9.1)	1 (2.8)	0.551
	Diplopia	2 (3.4)	1 (5.9)	1 (2.4)	0.504	1 (4.5)	1 (2.8)	1.000
	Facial redness or discoloration	2 (3.4)	2 (11.8)	0 (0)	0.082	2 (9.1)	0 (0)	0.140
	Fever	2 (3.4)	2 (11.8)	0 (0)	0.082	2 (9.1)	0 (0)	0.140

Duration of symptoms	12 [5–20.5]	10 [4.25–27.5]	14 [5–20.5]	0.638	12 [5–20]	10 [4.75–30]	0.852	

Symptom duration group	One week	14 (34.1)	7 (43.8)	12 (36.4)	0.650	7 (38.9)	12 (38.7)	1.000
	One week till one month	20 (48.8)	6 (37.5)	17 (51.5)		9 (50.0)	14 (45.2)	
	Above one month	7 (17.1)	3 (18.8)	4 (12.1)		2 (11.1)	5 (16.1)	

^*∗*^Chi-square test or independent sample *t*-test/Mann-Whitney *U* test COVID-19: coronavirus disease of 2019; DVT: deep venous thrombosis; Dx: disease; PTE: pulmonary thromboendarterectomy.

**Table 2:** Hospitalization characteristics of patients with mucormycosis.

Variable	Total; *N* = 59	Timeline		*P* value^*∗*^	COVID-19		*P* value^*∗*^
		Non-COVID-19 period; *n* = 18	COVID-19 period; *n* = 41		Not infected; *n* = 22	Infected; *n* = 37	
Hospitalization duration	1.12 ± 0.42	1.16 ± 0.51	1.10 ± 0.38	0.582	1.14 ± 0.47	1.11 ± 0.40	0.827
SPO2 on admission	96.07 ± 3.84	95.76 ± 5.61	96.20 ± 2.89	0.701	96.59 ± 5.05	95.75 ± 2.90	0.423
Temperature on admission	36.60 ± 0.37	36.77 ± 0.46	36.52 ± 0.29	0.047	36.67 ± 0.38	36.56 ± 0.36	0.243
Blood pressure on admission							
Systolic	125.52 ± 16.21	121.88 ± 13.84	126.98 ± 17.01	0.292	123.16 ± 13.92	126.73 ± 17.32	0.440
Diastolic	76.54 ± 13.05	72.75 ± 17.89	78.05 ± 10.43	0.172	75.32 ± 10.87	77.16 ± 14.14	0.621
Respiratory rate on admission	18.86 ± 2.56	18.59 ± 3.00	18.98 ± 2.39	0.605	19.33 ± 2.71	8.59 ± 2.48	10.296
Heart rate on admission	87.86 ± 15.24	93.94 ± 19.18	85.20 ± 12.50	0.041	90.23 ± 18.29	86.46 ± 13.17	0.363
Mucormycosis on admission							
Yes	42 (71.2)	13 (72.2)	29 (70.7)	**1.000**	12 (54.5)	30 (81.1)	**0.040**
No	17 (28.8)	5 (27.8)	12 (29.3)		10 (45.5)	7 (18.9)	
Mucormycosis diagnosis duration from admission	8.38 ± 6.02	7 ± 6.08	6.75 ± 4.65	0.738	7 ± 6.08	8.5 ± 6.09	0.738
Lung CT finding							
Ground glass opacities	24 (61.5)	0 (0)	24 (61.5)	**<0.001**	2 (22.2)	22 (73.3)	**0.015**
Interlobular septal thickening	9 (23.1)	1 (12.5)	8 (25.8)	0.653	0 (0)	9 (30.0)	0.085
Cardiomegaly	2 (5.1)	0 (0)	2 (6.5)	1.000	0 (0)	2 (6.7)	1.000
PNS CT							
Total performed	29 (49.2)	13 (72.2)	16 (39.0)	**0.025**	10 (45.5)	19 (51.4)	0.789
Mucormycosis or fungal infection	8 (27.6)	3 (23.1)	5 (31.3)	0.697	2 (20.0)	6 (31.6)	0.675
Mucosal thickening	15 (51.7)	4 (30.8)	11 (68.8)	0.066	3 (30.0)	12 (63.2)	0.128
Sinusitis	14 (48.3)	7 (53.8)	7 (43.8)	0.715	6 (60.0)	8 (42.1)	0.450
Bone erosion or destruction	6 (20.7)	3 (23.1)	3 (18.8)	1.000	1 (10.0)	5 (26.3)	0.633
Soft tissue mass	5 (17.2)	1 (7.7)	4 (25.0)	0.343	3 (30.0)	2 (10.5)	0.306
Soft tissue swelling	3 (10.3)	1 (7.7)	2 (12.5)	1.000	0 (0)	3 (15.8)	0.532
Orbital CT							
Total performed	8 (13.6)	0 (0)	8 (19.5)	0.092	2 (9.1)	6 (16.2)	0.697
or fungal infection	3 (37.5)	0 (0)	3 (100)	—	0 (0)	3 (50.0)	0.464
Sinusitis	4 (50.0)	0 (0)	4 (100)	—	2 (100)	2 (33.3)	0.429
Cellulitis	2 (25.0)	0 (0)	2 (100)	—	0 (0)	2 (33.3)	1.000
Proptosis	1 (12.5)	0 (0)	1 (100)	—	0 (100)	1 (16.7)	1.000
PNS and orbital MRI							
Total performed	1 (1.7)	1 (5.6)	0 (0)	0.305	1 (4.5)	0 (0)	—
Sinusitis	1 (100)	1 (100)	0 (0)	—	1 (4.5)	0 (0)	—
Scalp enhancement	1 (100)	1 (100)	0 (0)	—	1 (4.5)	0 (0)	—
Globe protrusion	1 (12.5)	1 (100)	0 (0)	—	1 (100)	0 (0)	1.000
Laboratory data							
White blood cell count	11.70 ± 5.77	11.82 ± 6.25	11.56 ± 5.44	0.908	11.80 ± 5.94	11.59 ± 5.80	0.923
Lymphocyte count	24.91 ± 20.25	2.40 ± 1.93	1.76 ± 1.19	0.397	2.21 ± 2.14	2.11 ± 1.31	0.910
Neutrophil count	70.55 ± 19.12	7.34 ± 3.39	7.93 ± 4.98	0.802	7.74 ± 5.18	7.42 ± 2.93	0.894
Hemoglobulin count	10.94 ± 2.37	10.73 ± 2.65	11.06 ± 2.24	0.659	10.56 ± 2.49	11.26 ± 2.28	0.330
Platelet count	281.76 ± 201.89	364.53 ± 285.15	225.31 ± 86.22	0.086	375.29 ± 258.53	202.25 ± 80.36	**0.016**
RT-PCR							
Positive	33 (58.9)	0 (0)	33 (80.49)	**<0.001**	0 (0)	33 (94.3)	**<0.001**
Ventilation							
Room air	37 (64.9)	11 (68.8)	26 (63.4)	0.239	17 (81.0)	20 (55.6)	0.113
Noninvasive or nasal mask	14 (24.6)	2 (12.5)	12 (29.3)		2 (9.5)	12 (33.3)	
Invasive and intubation	6 (10.5)	3 (18.8)	3 (7.3)		2 (9.5)	4 (11.1)	
Chest tube insertion	2 (3.8)	0 (0)	2 (5.1)	1.000	0 (0)	2 (5.7)	0.543
Operation							
Bilateral FESS	23 (39.0)	4 (22.2)	19 (46.3)	0.104	6 (27.3)	17 (45.9)	0.131
FESS + additional operation^*∗*^	19 (32.2)	7 (38.9)	12 (29.3)		11 (50.0)	8 (21.6)	
Rt FESS	12 (20.3)	5 (27.8)	7 (17.1)		3 (13.6)	9 (24.3)	
Lt FESS	3 (5.1)	0 (0)	3 (7.3)		1 (4.5)	2 (5.4)	
Outcome							
Discharge	28 (49.1)	8 (47.1)	20 (50)	0.433	12 (60.0)	16 (43.2)	0.124
Expired	21 (36.8)	8 (47.1)	13 (32.5)		4 (20.0)	17 (45.9)	
Relation of COVID-19 and mucormycosis							
Not related to COVID-19	15 (25.4)	9 (50)	6 (14.6)	<0.001	15 (68.2)	0 (0)	<0.001
During COVID-19	12 (20.3)	0 (0)	12 (32.4)		0 (0)	12 (32.4)	
Post-COVID-19 under 2 weeks	15 (25.4)	0 (0)	15 (40.5)		0 (0)	15 (40.5)	
Post-COVID-19 over 2 weeks	7 (11.9)	0 (0)	7 (18.9)		0 (0)	7 (18.9)	
Mucor onset before positive COVID-19	3 (5.1)	0 (0)	3 (8.1)		0 (0)	3 (8.1)	

^*∗*^Additional operations such as maxillectomy, orbital exenteration, and frontal lobectomy.

## Data Availability

The datasets generated and/or analysed during the current study are not publicly available because of patients details, but they are available from the corresponding author upon reasonable request.
